# Assessment of feedback modalities for wearable visual aids in blind mobility

**DOI:** 10.1371/journal.pone.0170531

**Published:** 2017-02-09

**Authors:** Aminat Adebiyi, Paige Sorrentino, Shadi Bohlool, Carey Zhang, Mort Arditti, Gregory Goodrich, James D. Weiland

**Affiliations:** 1 Department of Biomedical Engineering, University of Southern California, Los Angeles, California, United States of America; 2 Department of Biological Sciences, University of Southern California, Los Angeles, California, United States of America; 3 Department of Electrical Engineering, University of Southern California, Los Angeles, California, United States of America; 4 University of Southern California Eye Institute, Los Angeles, California, United States of America; 5 Psychology Service and Western Blind Rehabilitation Center, Veterans Affairs, Palo Alto, California, United States of America; 6 Department of Ophthalmology, University of Southern California, Los Angeles, California, United States of America; University of Ottawa, CANADA

## Abstract

Sensory substitution devices engage sensory modalities other than vision to communicate information typically obtained through the sense of sight. In this paper, we examine the ability of subjects who are blind to follow simple verbal and vibrotactile commands that allow them to navigate a complex path. A total of eleven visually impaired subjects were enrolled in the study. Prototype systems were developed to deliver verbal and vibrotactile commands to allow an investigator to guide a subject through a course. Using this mode, subjects could follow commands easily and navigate significantly faster than with their cane alone (p <0.05). The feedback modes were similar with respect to the increased speed for course completion. Subjects rated usability of the feedback systems as “above average” with scores of 76.3 and 90.9 on the system usability scale.

## Introduction

As of 2014, it has been reported that over 285 million people are visually impaired worldwide and of this population, 39 million are blind [1]. Visual impairment includes the subcategories of blindness (best vision of ≤20/200 in the better eye in the United States and <20/400 by the WHO) and low vision (<20/40 in the United States and <20/60 according to the WHO) and are due to a myriad of causes [2]. Among them the major causes are cataracts, age-related macular degeneration, diabetic retinopathy and glaucoma. Many blinding diseases are age-related and are considered incurable while others may have treatments that are limited in their effectiveness. The World Health Organization estimates that 65% or some 185 million people are over the age of 50 and population trends make combating disability due to vision-loss a pertinent issue. Within the United States population, the National Eye Institute estimates 3.5 to 5 million individuals are visually impaired, of whom more than 1 million are legally blind [3].

People with visual impairments, of all ages, are severely limited in their mobility and other activities of daily living that rely upon vision [4]. Orientation and mobility training improves the ability of individuals who are blind to navigate independently. The primary aid is the long cane; however, some individuals prefer a guide dog. In the United States, it is estimated that of the 1.1 million individuals who are blind 109,000 (10%) use a long cane and 10,000 (1%) use a guide dog [5]. Thus it appears that most (89%) individuals who are blind may not use any mobility aid. Electronic travel aids (ETAs) have been developed to increase capability beyond the white cane and guide dog. ETAs sense the environment (using a camera, laser, or sound signal) and provide non-visual signals (sounds or vibration) to alert the user to nearby objects. While ETAs date back some five decades [6], it appears that they are not routinely used by the vast majority and clearly more research and development is needed to make ETAs useable by more individuals who are visually impaired [7] (see references for a more complete discussion).

Early ETAs have been criticized as placing a burden on the user to interpret raw information. Basically, this criticism suggests that the user is over-tasked by having to respond to environmental stimuli (e.g. ambient sounds) as well as needing to decode the output of the ETA. An alternative approach is to reduce the information to simple commands, by having algorithms resident on the ETA process the sensor input. For example, an ETA with a camera could have an algorithm that processes camera data to locate a door, then the user can be guided towards the door with simple directional cues. Research has been conducted on how information gathered by an ETA should be provided to the user. While this literature is too extensive to summarize here, some studies are particularly relevant. The question of speech or auditory cues as output from an ETA has been examined [8]. Speech output was the preferred output medium, based on a questionnaire survey of ten well-educated and employed individuals with visual impairment. Speech output is also supported by a study a “Wizard of Oz” mobility device [9]. ETA output was also studied by comparing speech to virtual sound [10]. Virtual sound was shown to produce better performance when subjects performed a vibrotactile *N*-back task while guided along virtual paths without vision. However, producing virtual sounds is more difficult computationally (compared to generating words) and, to maintain fidelity of the stereo sound, requires users to wear headphones which block some or all ambient sound, thus limiting its practicality. Speech can be delivered via bone-conduction headphones that do not occlude ambient sounds. Currently it appears that determining “the best” (if there is a “best”) output medium will require additional studies which consider a variety of output mediums, ETA characteristics, environmental conditions, and personal preferences and characteristics of the user, among other variables.

Our group is developing a wearable visual aid (WVA) that computes the best traversable path in real-time using machine-vision principles [11]. In a previous study of a prototype WVA system, the WVA detected obstacles in the environment and computed a path around the obstacles, and transmitted directional commands, not raw data, to the user using body worn vibration motors. The main task of the user was to adhere to the computed path using only the simple cues provided by the WVA. The prototype WVA was shown to reduce collisions in the visually impaired when traversing an obstacle course. This study shows that vibrotactile cues also appear to useful for guiding navigation.

Based on the prior work in our lab and others, reviewed above, we can hypothesize that ETAs can be learned quickly and used effectively if simple, intuitive commands are provided to the user as guiding cues. The purpose of this paper is to report on a study comparing two types of ETA outputs (speech or tactile) in a group of blind test subjects. Most other studies of this type used blindfolded, sighted individuals and did not directly compare speech and tactile outputs. Prior research has specifically cited the lack of user-centered design as a barrier in the successful implementation of these devices by the visually impaired population [12–18]. And, for that reason our subject population included individuals who were blind.

In this study, we specifically focus on the critical aspect of maintaining the user on the preferred path via non-visual methods of feedback; specifically, the interface between the device and the user. The preliminary WVA study cited above involved only three subjects, traversing a single course multiple times, and utilized only tactile cues for guidance [19–20]. Orientation and mobility specialists often use verbal commands to direct their subjects in training, so in this study we explored the use of electronically delivered speech, like those that would be generated by a wearable visual aid. We also compared speech to “equivalent” vibrotactile commands. We explored vibrotactile commands as these will not interfere with the hearing of the visually impaired, as hearing is heavily relied upon for self-navigation. As previously noted, this research is part of a larger WVA project that seeks to use computer vision algorithms to predict clear paths and plan routes. The WVA software is still under development and is not yet sufficiently robust to reliably predict paths. Indeed, route planning and obstacle avoidance remains an active area of research in robotics and computer vision. Since the WVA is not yet robust enough to be used, we instead used a “human-in-the-loop” paradigm, similar to that used by Polacek [9] and others, to ensure that reliable directional cues were provided to the subject and to simulate the expected functionality of the WVA.

## Methods

### Mobility feedback systems

#### Audio mobility feedback system

The audio mobility feedback system (aMFS) is a tool developed by our group to assess speech cues for mobility through synthesized speech. Our design rationale was rooted in communicating navigational cues in as direct a ‘language’ as possible to minimize the amount of decoding our users will face. Although the use of virtual sounds in providing simple guiding cues has been demonstrated as superior to synthesized speech in minimizing cognitive load [10, 21], the infancy of its deployment to bone conduction headphones deemed it impractical for our purposes [22]. In addition, synthesized speech provides an expressiveness [18] that blind subjects familiar with common mobile platforms are already comfortable.

**Fig 1:**
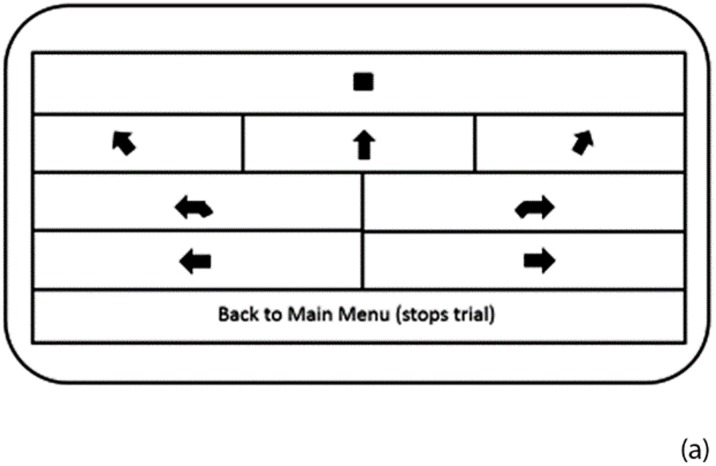
Custom android application provides audible commands for the Audible Mobility Feedback System. Front view of the custom android application showing commands implemented as buttons on a touchscreen. The outputs were audible commands delivered to the subject via bone conduction headphones.

The aMFS consists of bone-conduction headphones (GameChanger Innovations LLC) worn by the user and a custom android application to generate verbal commands under experimenter control. Bone-conduction headphones allow users to hear ambient sounds, which is important because visually impaired individuals are trained through perceptual learning to rely upon their hearing and other senses to enhance their mobility performance [23, 24]. The aMFS delivers speech commands to the user when an operator touches a virtual button on a touch screen (Fig 1). Eight commands included “forward”, “veer left”, “approaching left turn”, “turn left”, “veer right”, “approaching right turn”, “turn right” and “stop” were used. The duration of the commands was as follows: stop—0.75 seconds; approaching right/left turn– 2.53 seconds; forward—0.93 seconds; veer right/left—1.08 seconds; turn right/left—1.24 seconds. For testing reported here, the app was run on a Motorola XOOM MZ601 tablet and a dual-core Android 3.1 Operating System.

#### Vibrotactile mobility feedback system

The vibrotactile mobility feedback system (vMFS) is a collection of six vibration motors attached on individual points on a subject’s upper torso through a vest and activated by a push-button system. This system was intended to serve as a vibrotactile analog to the aMFS, providing the same eight commands through an array of six coin-shaped vibration motors which are eccentric rotating masses commonly used in cellphones and pagers and are also referred to as pancake motors.

The placement of the vibrotactile array was informed by other studies intersecting with our design constraints regarding portability and subject preference. Stimulation sites used by other studies include the tongue, hands and fingers, the waist and upper torso [25]. Although the hands and fingers are highly sensitive, it was ruled impractical due to the high usage of those areas during navigation with the white cane. The tongue was also similarly avoided for practical purposes, as our training protocol required our subjects to use their speech while navigating. Between the waist and the upper torso, the upper torso was chosen due to the number of distinct commands to be communicated and to provide a wide enough area so that each command could be discriminated clearly [26]. Our ultimate selection of a torso-based array is supported by studies showing not only its utility in a variety of mobile and strenuous environments [27], but also by the superiority of the back (upper torso) in pattern identification as compared to the forearm [28]; this study also showed that the type of vibration motor used did not affect pattern identification.

**Fig 2:**
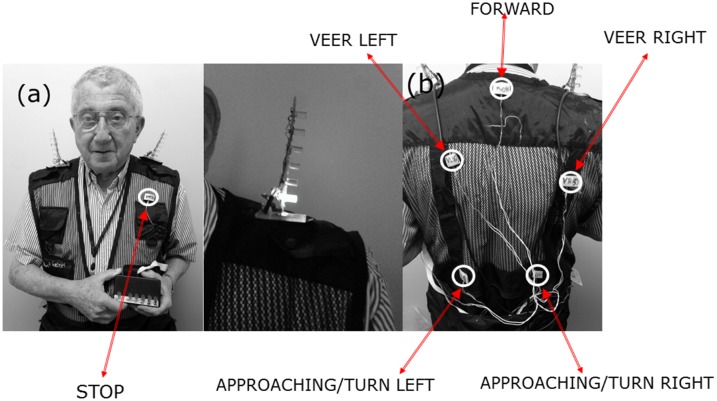
Vibrotactile mobility feedback system is a vibrotactile complement to the Audible Mobility Feedback System. (a) Arditti outfitted with the Vibrotactile Mobility Feedback System (vMFS) with an activated left turn command displayed on LED Array (centre) (b) Back view of vMFS showing placement of vibration motors on upper torso.

The coin vibration motors used in this experiment were manufactured by Yuesui (https://cdn.sparkfun.com/datasheets/Robotics/B1034.FL45-00-015.pdf). The motors are connected to a push-button microcontroller system that delivers commands to the subject when the researcher presses a button that activates the corresponding motor(s). The system was programmed using an Arduino^™^ ATMega development board and IDE environment. Eight navigational commands (corresponding to the eight speech commands of the aMFS) were encoded into the six-motor array as follows: forward—center back motor; stop—center front motor; veer left/right—upper shoulder; approaching left/right turn and turn left/right—lower back area (Fig 2). The duration of each vibrational pulse was 0.38 seconds. LEDs were connected in parallel to the motors and arrayed on the shoulder, to allow synchronization of the command and the subjects’ reaction, extracted from recorded video.

### Test subject demographics

Testing was conducted under a protocol approved by the University of Southern California Health Sciences Institutional Review Board (USC-HSIRB). A copy of the informed consent was provided to potential subjects to take home and discuss with their families, if they felt it was necessary. Once subjects indicated firm interest in participating, written consent was given after the forms were read to them and all their questions were answered. Subjects also consented to use of their health information for research by signing HIPAA authorization forms. The USC-HSIRB specifically approved all consent forms and procedures before they were administered to study participants.

Once enrolled, background medical information was obtained on their eye condition both from their ophthalmologist and from a questionnaire, under HIPAA regulations. All subjects had light perception or less, and therefore classified as totally blind with regards to functional vision. Subject code, age, gender and visual diagnosis are shown in Table 1.

**Table 1:** Subject demographics.

Subject	Age	Gender	Diagnosis of Vision Loss
**01**	50	M	Cytomegalovirus Retinitis
**02**	41	M	Advanced Glaucoma
**03**	55	F	Microphthalmia(Left)/Anophthalmia (Right)
**04**	47	F	Retinitis Pigmentosa
**05**	63	M	Cataracts
**06**	50	F	Diabetic Retinopathy and Glaucoma
**07**	69	M	Retinitis Pigmentosa
**08**	40	F	Detached Optic Nerve (Congenital)
**09**	64	F	Retinopathy of Prematurity
**10**	40	F	Optic Nerve Hypoplasia
**11**	69	F	Retinitis Pigmentosa

Eleven persons with severe visual impaired were enrolled in our aMFS experiment (mean age = 53.8 years). After a period of six months, ten out of our eleven former participants returned for our vMFS experiment (mean age = 53.5 years). The cohort of subjects were trained and tested identically for both systems.

### Training

Subjects were trained on the meaning of each command as well as on the expected response before they were tested. Training usually occurred on the same day as testing; however, two subjects had multiple sessions of training or participated in pilot experiments with the aMFS on separate days prior to the testing reported here. The pilot experiments consisted of guiding the visually impaired subject around a course for 3 minutes by an operator, who gave commands that were randomized for each trial. The course in the pilot experiment measured 5.18 m x 5.18 m, and was interspersed with 0.3 m-sized cones every 1.5 meters. The pilot experiment had no measurable effect on their performance (see Discussion).

Typical training for the main experiments included theory and practical based segments. The theory-based segment was where the researcher explained how the prototype worked and the meaning of each command. Subjects were given the opportunity to ask questions and told to repeat the commands as they heard them. The command set was given three times in random order, and once the researcher was satisfied that the subject had an understanding of what each of the commands meant, the practical training segment commenced. Subjects practiced in an environment different from that in which they were tested, until the researcher observed they were comfortable executing each command correctly at least three times consecutively, which usually took about three to five minutes. Once practice was complete, testing on the actual routes commenced.

### Testing

**Fig 3:**
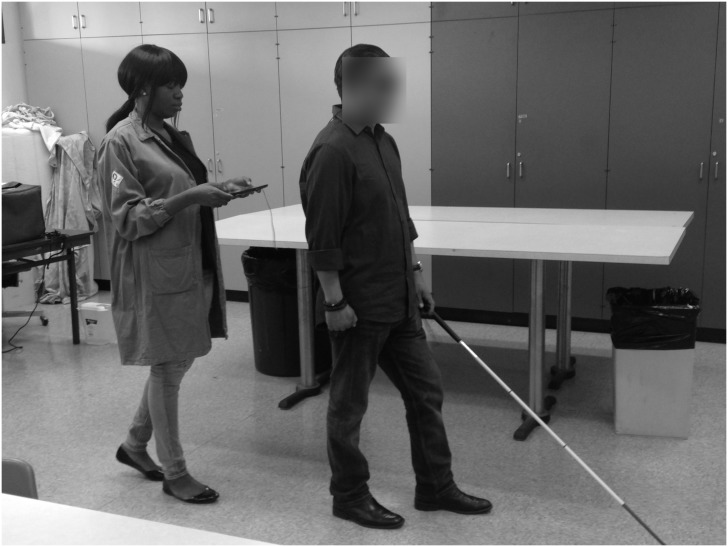
Indoor testing with the Audible Mobility Feedback System. Researcher guiding subject using the Audible Mobility Feedback System (aMFS) during an indoor testing session.

**Fig 4:**
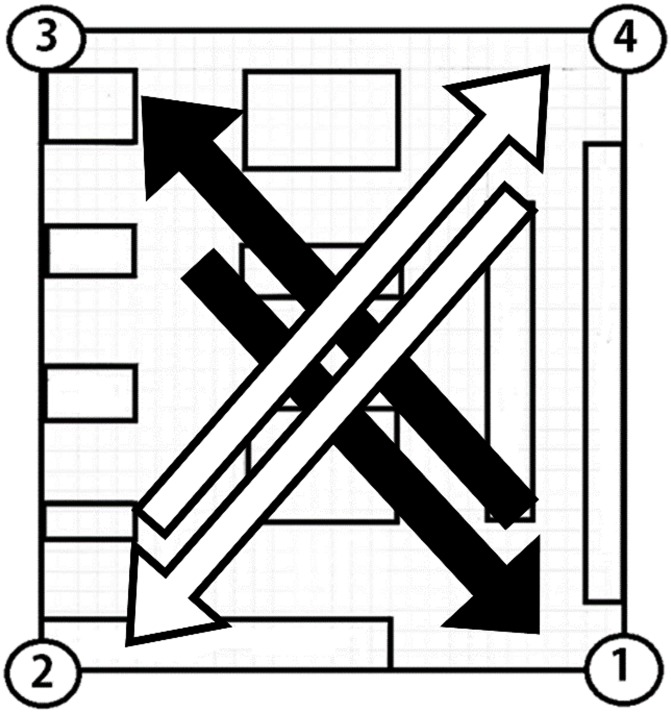
Layout of indoor mobility course. Schematic top view of the indoor mobility course used during experimentation. The numbered corners represent start and/or stop points for each trial. Each start point had a stop point at the diagonal corner of the room (direction of travel represented by arrows)

After training, subjects were guided through the indoor and outdoor mobility courses. Depending on the modality being tested, subjects used that MFS with their cane. The indoor setting was a classroom at the Braille Institute with tables, chairs and other obstacles (Fig 3). A top-view drawing of the room is shown in Fig 4. Only substantial obstacles like tables and countertops are represented; chairs were present during trials but were not substantial obstacles as they were pushed in towards the table.

**Fig 5:**
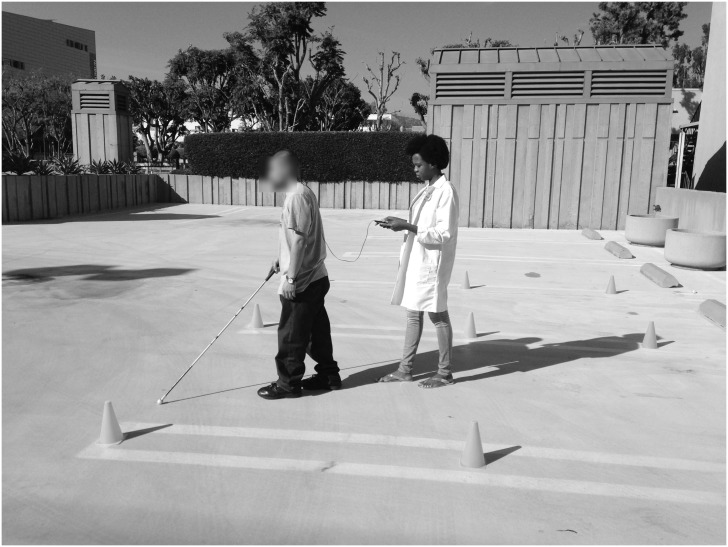
Outdoor testing with the Audible Mobility Feedback System. Subject being guided by the researcher using the aMFS during an outdoor testing session.

Starting points for the indoor setting were the four corners of the room. Subjects were asked to navigate diagonally across the room from one corner to the other, resulting in four different routes. As a control, subjects were asked to navigate these routes independently with their cane and their wayfinding skills. The outdoor setting consisted of an 8.53m by 6.40m course interspersed with 0.35m traffic cones, and subjects were guided around this course for a single three-minute trial (Fig 5).

For each modality, a subject was trained and tested entirely in one session. A testing session consisted of sixteen trials for the indoor setting (four control and twelve MFS), and one trial for the outdoor setting as described above, except for two subjects, who completed the MFS trials on one occasion, and the control trials on another.

The order of testing was alternated between the MFS with the cane, and the cane only. For example, if a subject had used the MFS and cane first and then their cane only, the next subject would test with their cane only first, and then the MFS and cane. Additionally, the indoor routes were alternated such that subjects were guided from point one to three, and then back. After this path was complete, subjects were guided from point two to four and back. The order of path organization was different between subject sessions. The outdoor route was always completed at the end of a test session. After all testing was completed, subjects completed a survey of their experience with the device. This was administered by someone other than the MFS operator, so that subjects could freely express perspectives of their experience.

Appropriate responses to commands, path tracking data, and the subjects’ reaction times were measured for both indoor and outdoor settings. Time to completion was measured only for the indoor setting since visible reaction was difficult to determine through video of the outdoor setting. The Android application with the aMFS recorded a time stamp for each command and each trial of the experiments was recorded by video camera. For aMFS, reaction time was measured by syncing the start of the experiment from the video (in which the operator made a clear “start” motion to aid in video content analysis) with the time-stamp of the first command of the log file. Subsequent commands were also time stamped in the log file, and therefore commands could be aligned with the video (based on the video time stamp) and reaction time calculated. Reaction time was determined as the time difference from when the researcher gave a command to when a subject visibly executed the command. If a subject did not execute the command at all, no reaction time was calculated. The expected compliance for “approaching turns” command was no reaction; therefore, no reaction time was determined for those commands.

For vibrotactile feedback, the onset of command was visible to the experimenter via the LED display, which allowed measurement of reaction time directly from the video time stamp. The log file or LED display also indicated the type of command, which allowed determination of compliance to that command, based on the video of the subject’s response. Path travel was estimated from video and used to generate heatmaps that showed the amount of time a subject spent in a given space. Percentage Preferred Walking Speed (PPWS) was calculated by taking the ratio of the speed of subjects using the mobility feedback system to navigate the obstacle course and their Preferred Walking Speed (PWS) [29]. PWS was established by measuring subjects’ average speed when navigating three unique routes at their own pace assisted by a sighted guide. Subjects were also given an exit survey that quantified their impression of the usability of the MFS (as described next) [30].

Statistically, our quantitative data was analysed using a paired t-test to compare the PPWS of subjects during their use of the aMFS and the vMFS in the indoor obstacle course. A Pearson product-moment correlation coefficient of the average subject performance was also calculated to determine if there was a learning effect with each iterative trial. Given the small sample size, an analysis of the within- subjects effect was computed using time to complete data to determine effect size.

### Systems usability assessment

The system usability score acts as a means to quantify how useable a system is. Ten questions are given to the user. Each question is rated by the user on a scale from 1 to 5, in which 1 corresponds to strongly disagreeing with the question, and 5 corresponds to strongly agreeing with the question (see supplementary material for the questionnaire). The questions are structured in such a manner that the equation below (Eq 1) can be used to calculate the system usability score based on the System Usability Scale (SUS) [30].

$$SUS=\left[\sum \left(Scores_{OddNumbers}-1\right)+\sum \left(5-Scores_{EvenNumbers}\right)\right]*2.5$$ (1)

The output of the SUS equation ranges from 0 to 100, which tends to be misread as a percentage [31]. Rather, the score has been shown as having a strong correlation to descriptive scales, similar to letter grades used in school (A, B, C, etc.) [32, 33]. Based on multiple studies, an SUS score of 68 would be considered above average, and anything below 68 is considered below average [33].

## Results

### Audio mobility feedback system

The percent compliance is shown in Table 2, and includes subject compliance to all commands. In the indoor setting, subjects complied on average 92.25%, and reacted to commands at an average of 1.47 seconds. They also navigated at 40.45% of the preferred walking speed using the aMFS, compared to 31.12% with their cane alone. Subjects performed comparably in the outdoor setting with an average compliance of 95.28% and an average reaction time of 1.66 seconds.

**Table 2:** Audible Mobility Feedback System results.

Subject	Average Indoor % Compliance	Average Reaction Time (s)	PPWS Control	PPWS MFS	SUS score
**01**	84.42%	1.79	35.4%	39.4%	95
**02**	93.92%	2.02	31.2%	39.8%	100
**03**	90.64%	1.46	41.2%	43.1%	55
**04**	85.89%	1.58	42.1%	43.0%	95
**05**	95.79%	1.73	25.1%	36.7%	85
**06**	95.88%	1.46	25.7%	37.8%	100
**07**	98.53%	1.12	45.6%	48.9%	100
**08**	82.02%	1.19	32.5%	50.6%	95
**09**	95.74%	1.32	15.1%	24.1%	80
**10**	96.05%	1.35	23.1%	39.3%	97.5
**11**	100%	1.17	25.3%	42.3%	97.5
**Summary**	92.25%	1.47	31.12%	40.45%	90.9

Using the aMFS and cane, subjects completed an indoor route at an average of 41.05s, in comparison to 62.86s using only their cane. This improvement in time to complete was found to be statistically significant for the control (M = 62.86s, SD = 40.46s) and the aMFS (M = 41.18s, SD = 10.50s) conditions; t (43) = 3.975, p = .000. Participation in the pilot experiment did not appear to affect performance. Subjects EB and RT-2 were included in the pilot experiment, and their performance (PPWS, average compliance, and reaction time) was within the range of the other study subjects (Table 2).

The effect size of the within-subjects comparison of navigating with the aMFS and the cane alone was computed to be r = 0.366 (paired sample correlations), with Cohen’s *d*s of 0.536 and 0.733 − using the control and pooled variances, respectively. The interpretation of these values [34] suggests a non-trivial medium effect. To rule out the potential of a learning effect using the MFS, a Pearson-product moment test was also performed for both the average compliance and reaction times across all subjects as a function of trial number, and no statistically significant correlations were found (p > 0.1).

Based on the SUS, the aMFS was scored at an average of 90.9 in its current condition, which can be interpreted as an “A” or excellent according to descriptive scales [32]; summary results for each subject are presented in Table 3. Subjects preferred regular commands to reassure them the system was online even if the command did not result in changing direction (for example, repeating the command “Forward” during an extended straight section of a route). Subjects also expressed interest about the future availability of the device, and commented on how much the device could benefit them in everyday life.

**Table 3:** Summary of system usability scores for audible and vibrotactile feedback systems by subject.

Subject	aMFS	vMFS
**01**	95	87.5
**02**	100	80
**03**	55	55
**04**	95	73
**05**	85	75
**06**	100	100
**07**	95	67.5
**08**	80	47.5
**09**	97.5	87.5
**10**	97.5	90.0
**11**	100	*
**Average**	90.9	76.3

*Subject 11 did not participate in the vMFS trial and therefore did not take the SUS survey

### Vibrotactile mobility feedback system comparison

**Fig 6:**
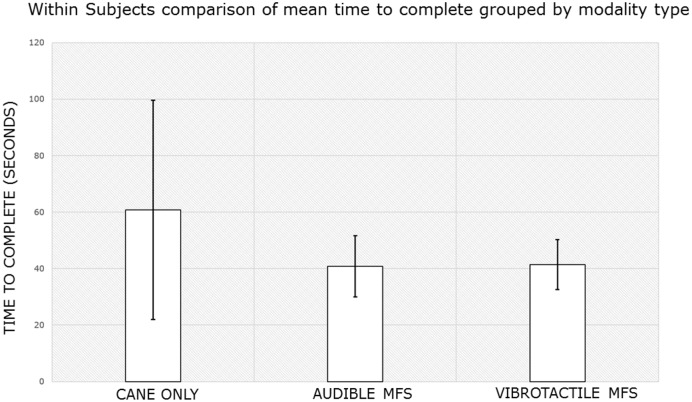
Mobility feedback system navigation showed equal improvement in completion time over cane-only navigation. Mean time complete grouped by modality type. Subjects completed the indoor course in less time using each mobility feedback system than with their cane alone. Improvement in performance the mobility feedback systems was equal, with no statistical significance (p = .677). Sample size are the ten subjects (n = 10) that participated in experiments with both the aMFS and vMFS.

On average, subjects complied 82.46% with commands and reacted to commands within 1.46s using the vMFS. Using a paired t-test, there was a statistically significant difference in the time to complete for the control (M = 60.80s, SD = 38.79s) and the vMFS (M = 41.45s, SD = 8.85s) conditions; t (39) = 3.477, p = .001. There is a medium-sized effect (r = 0.501) based on this within-groups comparison. They also navigated at 39.21% of their preferred walking speed compared to 40.45% with the audio MFS (Table 4). They rated the vMFS with an average system usability score of 76.3 (Table 3) which was less than the 90.9 score of the aMFS, although still above the average score of 68. Even though subjects preferred using the audio MFS based on their comments and the results of the SUS, there was no statistically significant difference in course completion times between the aMFS (M = 40.74s, SD = 10.84s) and vMFS (M = 41.45s, SD = 8.85s) conditions; t (39) = -0.419, p = .677. The paired sample correlations indicate a medium to large effect (r = 0.425) using the time to complete data. Fig 6 summarizes the mean time to complete within groups by modality type.

**Table 4:** Comparison of audible and vibrotactile mobility feedback systems.

Measure	Audible MFS	Vibrotactile MFS
**Average Compliance (%)**	92.25	82.46
**Average Reaction Time (s)**	1.47	1.46
**Average PPWS (%)**	40.45	39.21
**Average SUS score**^a^	90.9	76.3

^a^There were no significant differences on SUS scores between audible and vibrotactile mobility feedback systems.

**Fig 7:**
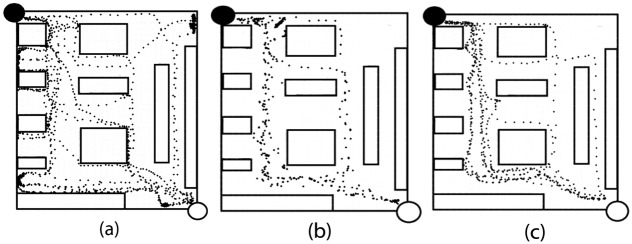
Mobility feedback systems improved efficiency of traversing obstacle course over cane-only navigation. Heatmaps showing trajectory plotted across all subjects in one of the navigated routes for the indoor mobility course. Concentration of black dots represents the amount of time spent in a space. White and black circles connote start and stop points, respectively (a) Control trial with subjects navigating with their cane alone (b) Subjects navigating with the aMFS (c) Subjects navigating with the vMFS.

Subjects’ travel routes as a function of time were represented using heatmaps for each indoor mobility task. The heatmaps depict one randomly selected trial of the three options for the same route (1 → 3) of each of the eleven subjects for the control and aMFS (Fig 7a and 7b) and ten subjects for the vMFS (Fig 7c). The efficiency of subject travel was improved using both mobility feedback systems (compared to the cane alone condition), with a limited amount of time spent in corners and in areas not essential to route completion.

## Discussion

Overall, our hypothesis that blind subjects will easily adapt to simple guiding cues for mobility was confirmed. The major findings of our study were: subjects traveled at a higher walking speed using either speech or vibrotactile feedback (compared to cane alone), adapted to both types of commands quickly, and completed routes more quickly using either mobility feedback systems. They traveled at a statistically significant higher PPWS in the indoor experiment (p < 0.05). Not only did their speed increase, the efficiency of their travel also drastically improved as shown by the heatmaps (Fig 7).

It is important to note that command compliance and reaction time were not statistically biased to a specific type of command (Table 5), but some trends in the data warrant further investigation. It would appear that subjects complied less with the approaching commands by preemptively executing the upcoming turns, probably due to their anticipation of the turns to come. Ideally, a positive compliance to an approaching command should not elicit a visible reaction from the subject, since the sole purpose is to warn them of an upcoming turn signal rather than prompt them to take action. This suggests that protocols should reinforce the meaning of commands. Also, based on anecdotal comments from subjects, some users may prefer not to have warning, and the final system should have the option to disable the warning commands. It also appears that subjects responded quicker to the stop command compared to other commands. This may be because of the simple nature of this command or because they may associate “Stop” with avoiding an imminent collision.

**Table 5:** Compliance and reaction time grouped by command type.

Command Type	Command Compliance	Reaction Time (seconds)
**Forward**	93.47%	1.49
**Veer Right**	93.64%	1.66
**Veer Left**	96.95%	1.56
**Turn Right**	97.11%	1.52
**Turn Left**	97.54%	1.53
**Stop**	91.26%	1.16
**Approaching Right Turn**	86.32%	*
**Approaching Left Turn**	77.55%	*

*The expected compliance for approaching turns was no reaction; therefore, no reaction time was determined for a positive compliance.

Once trained, subjects showed no statistically significant changes in reaction time or compliance with subsequent trials. This also demonstrates that subjects’ increasing familiarity with their environment did not positively affect their compliance or reaction time to commands. The training provided in this study was very rudimentary and usually done on the same day as testing, which suggests that this cohort of subjects quickly learned how to respond to these commands and use them effectively. As such, the system can be expected to be useful in a variety of unfamiliar settings.

When comparing compliance to commands and reaction time between the indoor and outdoor settings, we noticed no statistically significant differences (p > 0.05). This suggests that either feedback modality will be useful both indoors and outdoors. However, the outdoor environment was an empty parking lot with a low level of ambient noise. Feedback modalities should be tested in noisier environments, where the user may need to rely on their hearing more, for example, at a street crossing.

In testing subjects with the vMFS, we found that subjects reacted at about the same speed to commands as with the aMFS. However, they complied with commands at a lower rate than with the aMFS. It should be noted that the reaction time was measured from the start of the command. Since verbal commands necessarily took more time to deliver, the actual reaction time to a command is difficult to know. We can speculate that speech commands were understood faster once completed, but took longer to completely deliver whereas vibrotactile commands were sensed almost immediately, but required some time to interpret. The added task of interpretation may have led to the lower compliance. Despite the lower compliance rate, subjects navigated at a comparable PPWS as with using the aMFS (p > 0.1). In the exit survey, seven subjects expressed an interest in using the vMFS for street-crossing applications. These results indicate a selection of feedback modes could be used in the WVA for different tasks. Alternative positions for vibrotactile motors, such as on a glasses frame, should also be studied since wearing a vest is not always practical.

Subjects rated both systems highly usable (> 50%), however, they overwhelmingly preferred speech feedback over vibrotactile feedback. When probed about this difference in usability, subjects explained that they preferred the direct language over decoding the meaning of a vibration in a given region of the upper torso. This extra layer of mental processing quite possibly places extraneous mental load on the user, as they not only have to remember what the placement of each motor means, that is, a new language of sorts, but also how each command is meant to be executed. Further testing should be done to determine whether training could possibly minimize this mental load, so that decoding vibrotactile cues is as intuitive as speech. When technology permits consistent and successful virtual sound use with bone conduction headphones, it should also be explored in these environments to see if mental load could be further reduced. In general comments, several subjects stated a need for better electronic travel aids to assist in mobility.

Other researchers have studied the human interface of a navigation system for blind people. Polacek used a similar ‘Wizard of Oz’ approach to validate a set of speech-based navigation commands in a field study context [9]. Their goal was to conduct a pilot study to evaluate a generic Wizard of Oz system they had designed for mobile and ubiquitous studies. They employed eight humans ‘wizards’ to guide two blindfolded actors through a predefined route. While they were convinced that their setup was fully mobile, and their set of voice commands could be used for the follow-up study, they identified several usability flaws with their system. Comparatively, our study used one wizard to minimize variability in giving directions, and test subjects who were visually impaired.

Arditi’s findings [8] are consistent with our SUS results that indicate subjects prefer audible feedback for providing directional information. Their study surveyed user preferences and needs from a sample of ten well educated and employed subjects with light perception or less. They found that subjects would prefer speech as a means of communicating with their environment, interfaces that provide control, and the capability of verbally querying the system in lieu of interacting with a menu. While subject preference is important, and positively correlates with patient compliance, our goal was to quantitatively assess subject performance with these modalities. From this perspective, although our subjects may not prefer vibrotactile feedback, its utility for other applications where speech might not be an option (street-crossing, noisy environments) was validated. Its application could also be useful for deaf-blind subjects where speech may not be an option.

Our findings that speech cues will be effective in providing mobility feedback to the visually impaired are also consistent with the work of Havik [35]. That study compared the efficiency of different types of verbal information (route and environmental) provided by the Groningen Indoor Route Information System (GIRIS); an electronic navigation system designed to assist visually impaired (low vision and blind) travelers with wayfinding. Havik found that participants with low vision were most comfortable and showed the highest walking efficiency (PPWS) when walking routes with the GIRIS system, which provided verbal instructions en route. In contrast, the walking efficiency of subjects whose vision classified as blind was highest when using verbal guiding cues provided prior to embarking on the route. In comparison, our study specifically compares navigation across a room using either a cane with verbal guiding cues or a cane alone. However, both our study and Havik’s show the potential benefit of verbal feedback during mobility related tasks. This is also consistent with our discussions with orientation and mobility instructors, who use verbal cues to guide their students.

## Conclusion

The results from the current study are encouraging, as both efficiency and efficacy of travel significantly improved for most subjects using our “person-in-loop” test systems compared to using their cane alone (p < 0.05). As both feedback modes (speech and vibrotactile) were similar based on subject performance, providing both would allow the user the freedom to select based on their preference and/or environmental demands. According to the System Usability Scale, the preferred mode would be speech feedback, however, subjects indicated that they would favor vibrotactile feedback for street-crossing applications so they can also rely on their hearing without the competing speech output of the WVA’s bone-conducted speech. These results indicate the selection of feedback modes usable for the WVA may be task-dependent, a subject for future scientific inquiry.

The use of a sighted operator facilitated our experiments to effectively narrow the variable of focus to assess the feedback component of the WVA system. Going forward, an autonomous system for the blind will require robust computer vision algorithms to inform reliable path planning and wayfinding. These functions can be provided by increasingly capable portable imaging and computing systems, e.g. smartphones. As wearable, portable computing systems become more powerful, our results suggest that both speech and vibrotactile cues are viable feedback mechanisms for assistive travel aids to visually impaired travelers. Such systems should allow the user to specify the type of feedback mechanism based on their preference, the environment, and the current task.

## Supporting information

S1 File System usability scale. Full text of the ten-item Leikart-style scale developed by John Brooke to quantify subjective usability of a device. Text includes explanation of the questionnaire and a sample of the scale at the end of the document.
(PDF)

## Abbreviations

aMFS: Audible Mobility Feedback System
ETA: Electronic travel aid
MFS: mobility feedback system
vMFS: vibrotactile mobility feedback system
WVA: wearable visual aid
